# Total body irradiation—an attachment free sweeping beam technique

**DOI:** 10.1186/s13014-016-0658-y

**Published:** 2016-06-10

**Authors:** Petra M. Härtl, Marius Treutwein, Matthias G. Hautmann, Manuel März, Fabian Pohl, Oliver Kölbl, Barbara Dobler

**Affiliations:** Department of Radiotherapy, Regensburg University Medical Center, Regensburg, Germany

**Keywords:** Total body irradiation, Sweeping beam technique, Lung shielding, Dosimetry, 3D treatment planning

## Abstract

**Introduction:**

A sweeping beam technique for total body irradiation in standard treatment rooms and for standard linear accelerators (linacs) is introduced, which does not require any accessory attached to the linac. Lung shielding is facilitated to reduce the risk of pulmonary toxicity. Additionally, the applicability of a commercial radiotherapy planning system (RTPS) is examined.

**Material and Methods:**

The patient is positioned on a low couch on the floor, the longitudinal axis of the body in the rotational plane of the linac. Eight arc fields and five additional fixed beams are applied to the patient in supine and prone position respectively. The dose distributions were measured in a solid water phantom and in an Alderson phantom. Diode detectors were calibrated for in-vivo dosimetry. The RTPS Oncentra was employed for calculations of the dose distribution.

**Results:**

For the cranial 120 cm the longitudinal dose profile in a slab phantom measured with ionization chamber varies between 94 and 107 % of the prescription dose. These values were confirmed by film measurements and RTPS calculations. The transmittance of the lung shields has been determined as a function of the thickness of the absorber material. Measurements in an Alderson phantom and in-vivo dosimetry of the first patients match the calculated dose.

**Discussion and conclusion:**

A treatment technique with clinically good dose distributions has been introduced, which can be applied with each standard linac and in standard treatment rooms. Dose calculations were performed with a commercial RTPS and should enable individual dose optimization.

## Background

Total body irradiation (TBI) plays a prominent role especially in the myeloablative conditioning prior to hematopoietic stem cell transplantation [1]. Many different schemes regarding the total dose, the fractionation and the dose rate are reported [2, 3]. However, 12 Gy in 6 fractions on 3 days is a very common myeloablative condition scheme [3, 4]. From 1995 to 2013 at our department different schemes have been applied [5] using a sweeping beam technique as described by Müller [6]. This technique not only used a gravity oriented shaped filter to compensate the effects of inverse square variation of the fluence with distance as it has later been investigated by Chui et al. [7], but also allowed the application of a set of lung shields close to the collimator.

The aim of this study was to establish a treatment procedure with similar parameters, when the linacs which had been employed for this sweeping beam technique had to be replaced [8, 9]. No accessory should be attached directly to the machine to avoid a certification process for in-house developed equipment [10]. Lung shielding should be facilitated to reduce the pulmonary toxicity [2, 11] as it was possible with the former technique. Abandoning of the gravity oriented accessory was a precondition to enable the calculation of the dose distribution with a commercial RTPS in clinical routine [12]. Although the application of a commercial RTPS is still quite uncommon in TBI with Linacs, as the technical conditions cannot be modelled for many of the applied techniques, single cases have been reported earlier [12–15]. In the recent years further adaptions of RTPS for TBI have been presented [16, 17].

This study presents the measurements required for the introduction of the new technique and the results of the in-vivo dosimetry of the first plans.

## Methods

### Linac, couch and patient positioning

Two linacs of type Elekta Synergy™ with Agility™ head (Elekta Ltd., Crawley, UK) and photon energies of 6 and 15 MV and electron energies of 6, 8, 10, 12 and 15 MeV were used for this study. Both linacs have been matched [18] and conform the requirement of a back-up concept, guaranteeing the completion of the treatment in time in case of machine breakdown [11, 19].

**Fig. 1:**
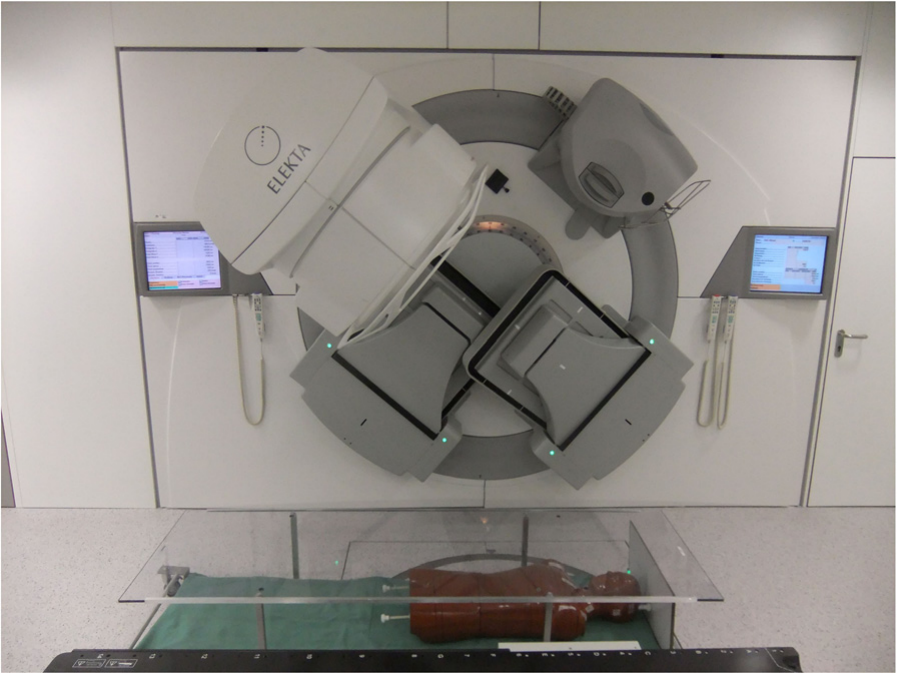
Linac in starting position and patient setup (Alderson phantom) on the couch

The patient is positioned on a low couch on the floor in supine and prone position, the longitudinal axis in the rotation plane of the gantry (Fig. 1). The positioning is supported by a soft mask allowing free air flow in the prone direction. The couch top is located 117.5 cm below the isocenter. A plate of Makrolon® polycarbonate of 10 mm thickness is placed on a stand above the patient to reduce the buildup effect in the patient [11]. The distance between the couch and the polycarbonate plate is 33 cm. The plate also serves as tray for the lung shields. The top of the patient’s head is always 60 cm from the vertical isocenter plane and is represented by the longitudinal position *l* = 0 cm. The dose reference point was defined on the vertical axis through the isocenter in the middle of the diameter of the patient or phantom at *l* = 60 cm. In most cases this is close to the umbilical transverse plane as a quite common reference point [19–21]. The position of the feet depends on the patient’s body length.

The low diameter in the cervical region is partially compensated in prone and supine positioning by a bolus of plastic modelling mass.

### Treatment fields

The photon beam energy chosen for TBI is 6 MV as rather common [11]. The main dose contribution is given by rotational fields (arcs) alternating from 310° to 70° clockwise and reverse. A collimator angle of 90° ensures a constant field width of 10 cm at the isocenter in the sweeping direction limited by the solid jaws. The multileaf collimator is set to an opening of 40 cm to exceed the couch width. A number of eight arcs per patient position and a dose rate of 300 monitor units (MU) per minute has been chosen to achieve a low mean lung dose rate [21] which has been discussed as a parameter to reduce pulmonary toxicity [20, 22–24]. For the compensation of the effects of inverse square variation of the fluence with distance, additional fields are required in the cranial and caudal direction, partially with wedge, the toe directed to the more distant areas. The parameters of these distance compensating fields are given in Table 1. The MU are kept constant as the diameters of the heads and legs vary less than in the trunk region.

**Table 1:** Parameters of the fixed beam fields (according IEC 61217 [36])

Field	Gantry Angle	Collimator Angle	Wedge	X1	*X*2	Y1	Y2	Monitor Units
Cranial	330°	270°	60°	−16.0	16.0	−14.5	14.5	316
Caudal 1	36°	90°	No	−16.0	16.0	−8.0	20.0	60
Caudal 2	36°	90°	60°	−16.0	16.0	−15.0	15.0	268
Caudal 3	36°	90°	No	−16.0	16.0	−15.0	20.0	50

Patients, whose lungs are shielded, are treated with electron fields in the shielded region to achieve the full dose to the thoracic wall. The energy of the electron fields is chosen depending on the thickness of the thoracic wall. The electron applicator 25 × 25 cm^2^ is equipped with individually shaped apertures and is applied in source skin distance of 110 cm.

### Measurements, phantoms and dosimeters

A solid phantom of water equivalent material (RW3, PTW Freiburg, Germany) was composed of slabs of 1 cm to cuboids of 30 cm width, 90 cm length, and 11–29 cm height, thus fulfilling minimum dimension requirements [20, 21]. From position *l* = 135 cm to higher values, which represents the region of the legs, the height was 11 cm constantly. This phantom was used for absolute dose measurements at the dose reference point, for profile measurements at a depth of half height, and for measurements of the lung shield transmission. Measurements in the phantom were performed using a Unidos dosimeter (PTW, Freiburg, Germany) with an ionization chamber (IC) M23332, a Roos chamber and Gafchromic™ EBT3 films (Ashland Inc., Covington, KY, USA). The films have been scanned by an Epson Perfection Scanner V700 Photo (Seiko Epson Corp., Nagano, Japan) and evaluated as described by Maerz et al. [25].

The results of the measurements in the stack phantom were used to create tables for calculations of the MU and were compared to the RTPS calculations.

A male Alderson phantom (RSD Inc., Long Beach, CA, USA) equipped with Gafchromic™ films was applied for final control of the dose distribution.

For in-vivo dosimetry a set of diode detectors (Isorad-p 6-12 MV, Sun Nuclear Corporation, Melbourne, FL, USA), suitable for TBI dosimetry [26], in combination with a Multidos electrometer and Multisoft software (PTW, Freiburg, Germany) has been calibrated against the Unidos dosimeter in the phantom of 21 cm of RW3 material in the TBI irradiation geometry. The in-vivo measurements were performed for the first ten patients with total doses of 4 Gy, 8 Gy, 10 Gy, or 12 Gy in the following points: on the forehead, neck ventral midline, chest midline, ventral and dorsal projection of the reference point, abdomen midline, ventral thigh and ankle. Temperature corrections were performed according the manual [27], which describes a variation of 0.3 % per degree Celsius. The measured values were compared to the calculations of the RTPS.

### Radiotherapy planning

Dose calculations were performed using the collapsed cone algorithm implemented in the Oncentra® External Beam planning system, version 4.3 and 4.5 (Elekta Ltd., Crawley, UK) which calculates dose to medium, introducing some workarounds:

- The patient is treated in supine and prone position. Although calculations on the supine study neglect changes in the body geometry induced by the prone positioning, it is more descriptive to have the complete calculations in one study.
- Rotational fields in the RTPS are approximated by stationary fields separated by 5° gantry difference. At the large distance of the patient this results in calculation artefacts. Consequently we always calculate five arcs with different starting points shifted 1°, thus getting a resolution of 1°. The MU are adjusted for the actually delivered eight arcs.
- Dose calculation in Oncentra is only possible for beams with the central axis intersecting the patient outline. To overcome this restriction, the patient outline is extended in cranial and caudal direction with density of air.
- In Oncentra absorbers can be modelled in accessory tray only and not on the distant polycarbonate plate. Therefore doses to the lung and thorax region have to be corrected manually when shields are applied.
- As the polycarbonate plate above the patient is not part of the CT scan, it has to be modelled different: a simple and fast procedure is adding a flap of 1 cm to the patient outline [15].
- The actual CT scanner Somatom Sensation (Siemens, Erlangen, Germany) is designed for maximum scan lengths of 150 cm. For larger patients the last scanned slice in the legs must be extended to the patient’s full body length by tools of the RTPS and therefore does not represent the actual geometry of the feet. Using a whole body spiral CT scanner (commonly used for primary diagnostics of poly trauma patients [28]) avoids this workaround.

Although the lung shields cannot be calculated for rotational fields, the RTPS is used for the generation of the blocks. A gantry position of 350° was found as mean value of entry and exit of the arc over the lungs. At this position block contours are generated by the radiation therapist. The dimensions are adapted to actual position on the polycarbonate plate.

Lung shields are only applied for a reference dose larger than 8 Gy. They are used as partial shield to reduce the total dose to the center of the lung *D*_*t,Lu*_ to 3.5 Gy in supine and prone position each, giving a total dose of 7 Gy for therapy regimen of 10 and 12 Gy. To evaluate the transmittance dependent on the material thickness of the alloy MCP96 three shields of 5, 10 and 20 mm were combined to blocks up to 35 mm in steps of 5 mm. The measurements were performed in the water equivalent phantom at a thickness of 21 cm.

The electron fields for the shielded region of the thoracic wall are treated once a day, delivering a total dose *D*_*e*_ of 3 Gy for the 10 Gy patients and 5 Gy for the 12 Gy patients respectively. The electron fields are planned with the RTPS Oncentra. The applicability of the Monte Carlo code in Oncentra has been evaluated earlier [29, 30]. The calculated percentage dose of the electron fields *p*_*e*_ in the center of the lung is used to calculate the thickness of the lung shields for the photon arc fields.

The thickness of the lung shields is derived from the dose calculated by the RTPS for the rotational fields without shields *D*_*x,Lu*_ and the dose contribution of the electron fields. The transmittance *T* is then given by Eq. 1:

1 $$ T=\left({D}_{t,Lu}-{D}_e\ast {p}_e\right)/{D}_{x,\;Lu} $$

## Results

### Profiles and absolute dose measurements

**Fig. 2:**
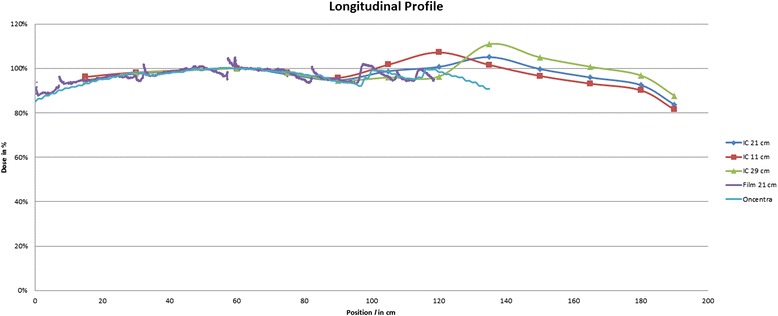
Profiles along the main axis measured with ionization chamber in a cuboid phantom for three different heights. Starting from position 135 cm to the right the height is 11 cm in all three cases. The dose is normalized at position 60 cm. For the standard height of 21 cm a measurement with Gafchromic™ film and the RTPS calculation are demonstrated

Figure 2 shows the profiles in the cuboid phantom for three different heights, the reference height (21 cm) and the intended minimum (11 cm) and maximum (29 cm) heights with an identical height of 11 cm for positions *l* ≥ 135 cm as described above. The normalization point is at position *l* = 60 cm. Down to position *l* = 120 cm which includes the head and trunk region, all values except one—at position *l* = 15 cm for a height of 29 cm at 94 %—are within 95–107 %. Only for the most distant position at *l* = 190 cm the dose drops below 90 %.

The longitudinal profiles in the reference phantom (height 21 cm), measured with Gafchromic™ film and calculated with the RTPS are included in Fig. 2. Spikes appear at the borders of the single films as artifacts at the film edges. Additionally, profiles in different depths have been determined with films, showing a very similar behavior.

The absolute dose in the reference point has been determined in the same phantom, using stacks of heights from 11 to 29 cm in steps of 2 cm. Table 2 shows the normalized number of MU referring to the reference stack of 21 cm.

**Table 2:** Dependency of the number of MU for a given dose value on the phantom height

Stack height in cm	11	13	15	17	19	21	23	25	27	29
Normalized number of MU	95 %	95 %	97 %	98 %	99 %	100 %	101 %	103 %	104 %	106 %

### Lung shields and dose rate

**Fig. 3:**
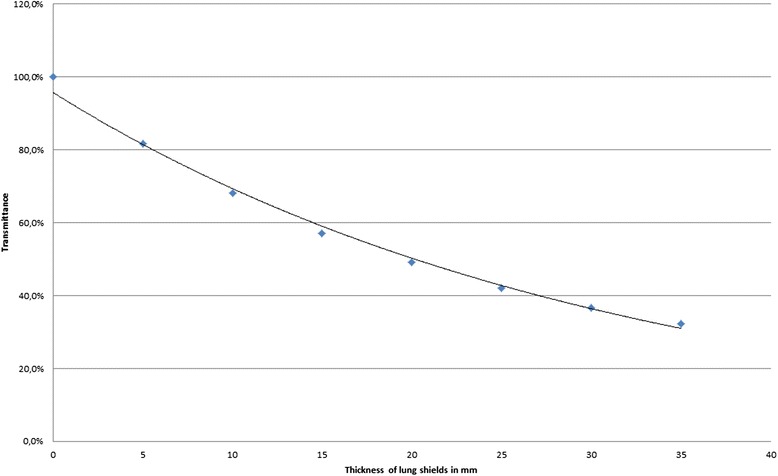
Transmittance of the lung shields as a function of the thickness of the MCP96 material. The dots represent the measured values, the line an exponential fit

The measurement of lung shields of different thickness of the MCP 96 material shows a range of the transmittance from 80.8 % at 5 mm thickness to 32.0 % at 35 mm. Figure 3 demonstrates the transmittance as a function of the thickness. When the arc field passes the chamber in the reference stack the dose rate is 0.55 Gy/min for unshielded fields. Taking the total time into account, to deliver the arc beams, including the time to turn the patient from the prone to the supine position, the average dose rate is about 0.04 Gy/min.

### Dose calculations and Alderson and in-vivo measurements

The MU calculated by the RTPS and the table showed good agreement within 0.7 % ± 2.1 % (sample standard deviation).

**Fig. 4:**
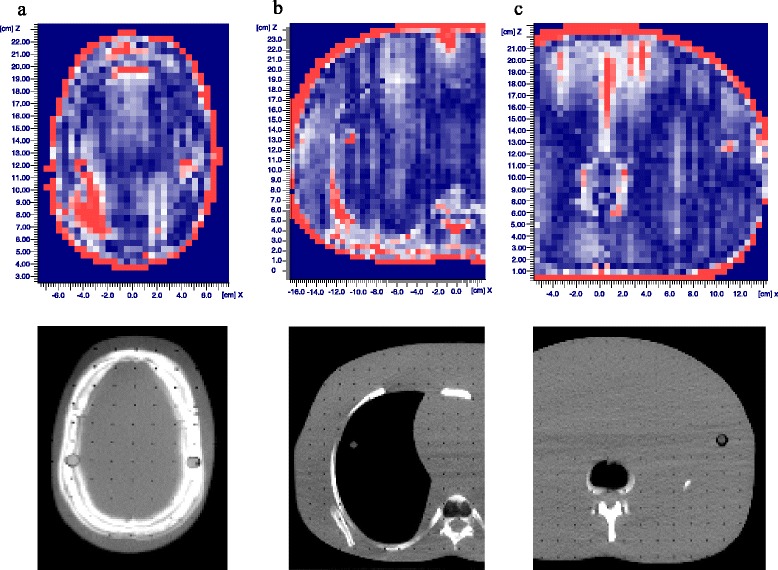
Gamma evaluations of three Gafchromic films in the Alderson phantom (**a** head region, **b** thorax, **c** abdomen) compared to the RTPS calculations. Read pixels do not fulfill the gamma criterion (3 mm, 3 %). Below the corresponding CT slices

The films in the Alderson phantom were compared to the corresponding slices of the RTPS calculations. Figure 4 shows the gamma evaluations (3 mm, 3 %). In the red pixels the gamma criterion is not fulfilled. The red margin emerges from the flap surrounding the phantom in the calculation. The positions of the bores in the phantom slices, which are used for the connecting rods appear also in red.

**Fig. 5:**
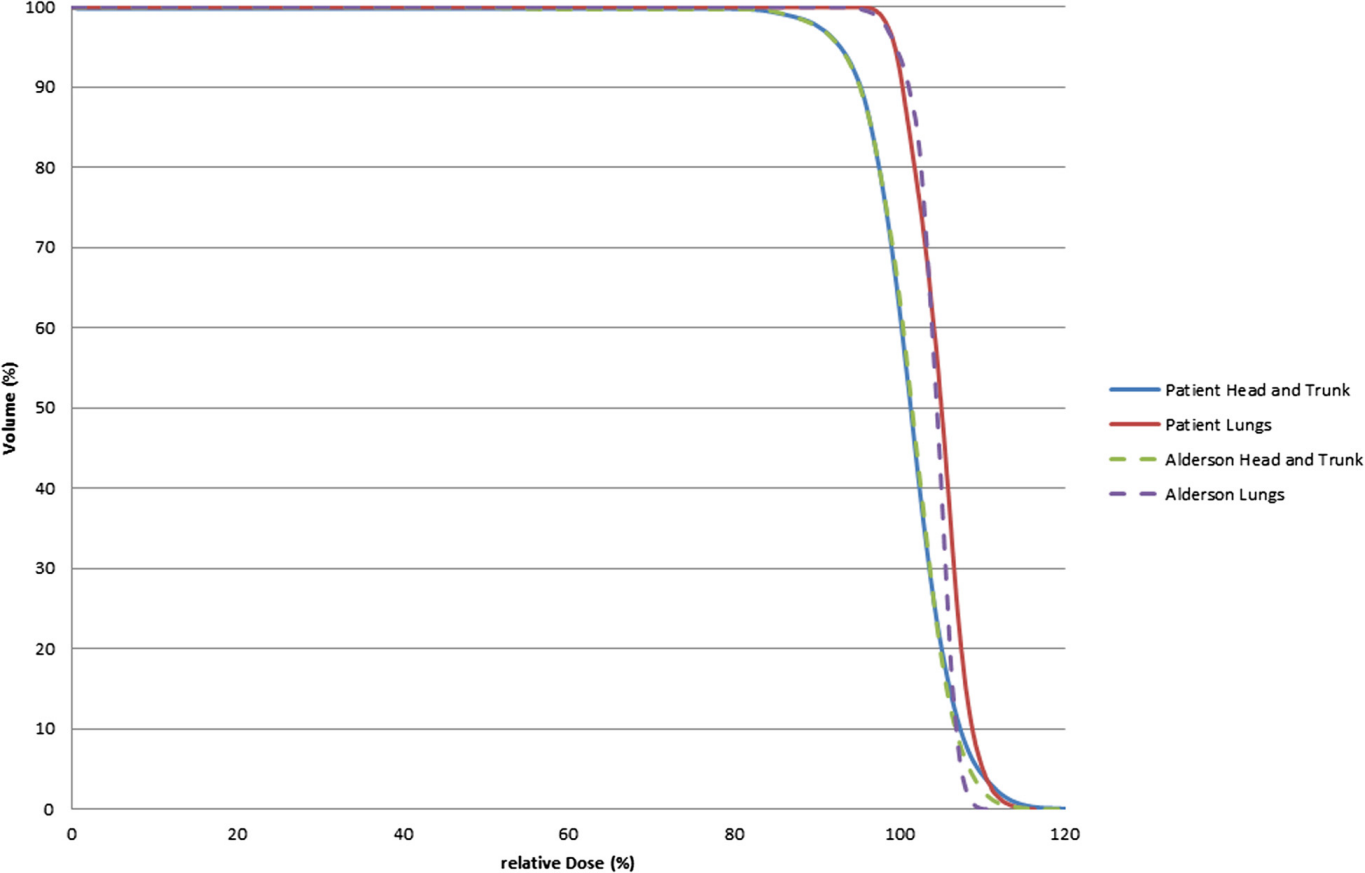
DVH of the Alderson phantom and a representative patient, containing the lungs as organs at risk and the combined head and trunk region

Figure 5 presents dose volume histograms (DVH) of the inhomogeneous Alderson phantom—which lacks the limbs—and of one representative patient with similar dimensions. Equivalent regions have been marked for comparison: The head and trunk region which for the Alderson phantom is the whole outline, and the lungs. The lungs have been combined to one volume as described by the German guideline [21]. The mean dose to the lungs is about 3 % higher than the mean dose to the complete phantom outline.

Table 3 contains the average and standard deviation of the measured dose values of the in-vivo dosimetry. Comparing these values to the results of the RTPS the deviation is 0.5 ± 4.7 %. The values for the legs were not considered in this comparison, due to the short scanning length of the CT scanner. The measurement points “reference ventral” and “reference dorsal” do not represent the reference point, but are only its vertical projection to the skin. Therefore the values are higher than the reference dose.

**Table 3:** Average and standard deviation of the in-vivo dosimetry for 10 patients

	Forehead	Neck ventral	Chest midline	Reference ventral	Reference dorsal	Abdomen	Thigh ventral	Ankle
Dose in Gy	2.01 ± 0.06	2.00 ± 0.05	2.04 ± 0.09	2.11 ± 0.08	2.04 ± 0.06	2.10 ± 0.10	2.12 ± 0.17	2.04 ± 0.10

## Discussion

The irradiation technique is simple and does not require any accessories except of the couch on the floor with the Makrolon® plate on top. Each standard linac is suitable for the technique. No additional modules for variable MU per degree are necessary. Treatment rooms of standard size can be used as it has also been pointed out recently [17, 31] for a sweeping beam technique with modulated arcs. The RTPS is essential only for the electron field calculations, and can be relinquished for treatments up to 8 Gy total dose. This has been verified by the very good agreement of the number of MU calculated by the RTPS and by the table. However, optimization of the dose distribution is possible by variation of the field parameters or additional fields and will further be investigated. The calculations should be possible with each RTPS, which allows rotational fields at larger distances. Nevertheless, commissioning for these non-standard conditions must be performed.

It has been stated in the section about radiotherapy planning that the planning system cannot handle the lung shields. Therefore a cumulative DVH of the real lung dose cannot be presented for the patient groups with 10 Gy total dose or higher. For patients without lung shields the DVH of the Alderson phantom and of one representative patient (Fig. 5) demonstrate a dose distribution to the body which is in the range described by Quast [11]. The mean dose to the lungs is higher for about 3 % in the phantom and similar in the patient due to the lower attenuation of the lung tissue. The calculated values are supported by the 2D film verification (Fig. 4).

It has been mentioned in the material and methods section that the dose rate has been chosen to achieve similar results as with the former technique regarding the lung toxicity. Furthermore effects of the dose rate on renal dysfunction have been discussed [32]. Recent publications confirm the pulmonary toxicity of TBI [2, 33, 34], but Sampath et al. doubt the dependency on the dose rate [35]. Consequently a large variety of applied dose rates has been reported [3]. Our current average value is close to the lower end of the reported range leading to total treatment times of about one hour per fraction or even more, if lung shields have to be positioned. However, reducing the number of arcs and increasing the delivered dose rate is possible to shorten the treatment times to about half an hour.

The in-vivo measurements with diodes are well in the range of accepted discrepancies between calculated and measured dose of the survey of Giebel et al. [3] of up to 10 %. Ramm et al. [26] have used the same type of detectors for over ten years in TBI treatments. However, they report an influence of less than 0.5 % of the temperature on the measured value and neglect it, which is not in accordance with our observations.

## Conclusion

We have established a treatment technique for TBI with standard linacs in standard treatment rooms. Having similar parameters as with the former treatment machines, the current treatments can build on an experience of nearly two decades in our department. Abandoning any attachments allows the commissioning of the method without additional certifications and enables the calculation of three-dimensional dose distributions with a commercial RTPS.

## Abbreviations

DVH, dose volume histograms; IC, ionization chamber; Linac, linear accelerator; MU, monitor units; RTPS, radiotherapy planning system; TBI, total body irradiation
